# 

**Published:** 1912-03-16

**Authors:** 


### Notes and Queries.

**(Correspondence and Enquiries Invited.)**

POSITION OF MALE NURSES UNDER THE ACT.

"Male Nurse" writes: Noticing in the current issue of your paper the article on the National Insurance Act department, and the offer of practical counsel contained therein, I should be greatly obliged if you would inform me what status the male nurse has under the Act. I have studied it fairly closely from various points of view, but owing to the intricate nature of the various conditions, have been unable to come to any definite conclusion. Doubtless there are several in the same "fix."

[No special provision is made under the National Insurance Act for male nurses. As employed persons, if their remuneration is not greater than £160 per annum, they will have to contribute 4d. weekly while in employment. This contribution will be made as a deduction from the wage, and the employer will have to make a payment of 3d. on behalf of each such nurse. If the wages are paid by an institution it would seem that the institution will be liable for the payment of the contribution, even though the nurse is attending a patient privately. If, however, the fees are paid by the patient to the nurse direct, the patient will be liable as the employer. We propose to deal with the variations of the standard benefits as allowed by the Act, as they are best suited to the requirements of male nurses, in the course of our articles.—Ed., The Hospital.]

### Buildings Contemplated and in Progress.

Hunstanton.—Additions and alterations are proposed to the Hunstanton Convalescent Home. Messrs. Macalister and Skipper, of Cambridge, are the architects. Tenders are now being considered by the committee.

Llandudno.—At a meeting of the Nicol Memorial Cottage Hospital authorities it was decided to erect an operating theatre at an estimated cost of £1,500.

Maghull.—Messrs. Briggs, Wolstenholme and Thorneley, of Liverpool, are architects for the proposed epileptic colony for 200 at Maghull for the Liverpool Select Vestry and the West Derby Board of Guardians. The architects' estimate of the cost was £46,200, and a tender of £44,685 has been accepted.

Ripon.—The Governors of the Dispensary and Cottage Hospital are contemplating extensions at a probable outlay of £2,000.

Sheffield.—A committee has been appointed by the Guardians to consider the question of providing additional accommodation at their hospital.

Whitby.—The Guardians invite schemes and tenders for heating certain wards, etc., on low-pressure system at the workhouse.

Wimbledon.—A tender of £1,846 is recommended for acceptance for enlarging the administration block at the Town Council's isolation hospital.

### Florence Nightingale Memorial.

The following contributions have been received during the past week by Mr. G. Q. Roberts: Lt.-Gen. H. Kent and the Duke of Cambridge's Own Middlesex Regiment, £50; the Queen's Bays, £18 10s.; collection at Norfolk and Norwich Hospital and Jenny Lind Infirmary, Norwich, £10 10s.; Adeline Duchess of Bedford, £10; Sir George Barham, £5 5s.; Hon. Richard M'Bride, £4; Frederick S. Clarke, Esq. (annuities), £3 3s.; Army Service Corps, Aldershot (in addition to £12 5s. 7d. already received), £2 4s. 6d.; C. S. Newton, Esq., £2 2s.; Lt.-Gen. Sir H. L. Smith-Dorrien, K.C.B., £2 2s.; W. P. Ker, Esq., £2 2s.; 39th Battery R.F.A., £2; collection by Miss Hamilton at Studley College, £1 6s. 3d.; staff of Torbay Hospital, Torquay, £1 1s.; Mrs. Register, £1; Sir William B. Richmond, £1; Major C. E. Higginbotham, £1; Mrs. Alfred Hill, 12s.; Miss E. M. Jones, 10s.; Anon., 10s.; Rev. J. Ridley, 5s.

### Appointments.

Mr. Harold H. Tanner, M.B., B.S. Lond., has been appointed assistant surgeon to the Evelina Hospital for Children, Southwark.

Mr. S. A. Ballantyne, F.R.C.S., has been appointed senior medical officer to the Coventry Workhouse. Mr. Ballantyne, who graduated M.B., Ch.B. at Edinburgh in 1900, has held the post of surgeon to the Coventry and Warwickshire Hospital.

### The Week's Novelties.

### KEROL DISINFECTANT.

No disinfectant can be highly commended solely because its strength, as gauged by a well-known laboratory test, is represented by a high number; for hospital workers several other considerations have to be taken into account. The Rideal-Walker test has been severely criticised, but in spite of its difficulties and its fallacies it still remains a factor on which manufacturers lay a certain insistence. As regards this particular disinfectant, Kerol, the manufacturers are able to boast a high coefficient against carbolic acid, and we may point out that the result of a searching inquiry into the question of these coefficients was to modify considerably the relative positions of many well-known disinfectants. But in the case of Kerol its position was if anything improved, and the figure on the newer scale—namely, 7.7—places it well in the front rank as regards its strength compared with carbolic acid. Kerol possesses many other qualities which should appeal to the medical and nursing professions. We may cite the ease with which it is miscible with water, either hot or cold, to form a uniform but opaque solution which is non-irritating to the skin and which is, moreover, compatible with soap, so that the cleansing and the sterilising of the hands can be conveniently carried out together. The disinfecting fluid itself is a homogeneous liquid of a pleasant odour and has no tendency to separate out into constituents of different specific gravities. It is of a low toxicity, and is in practice a very safe antiseptic to place in the hands of midwives, and is also one which does not stain permanently or damage ordinary fabrics. Kerol can be obtained combined in soaps, powders, etc., for various purposes, and the merits of these as compared with the corresponding preparations of other antiseptics are of the same high order as those of the liquid disinfectant itself. An important question, especially for hospitals, is that of price, and in this matter the manufacturers, Messrs. Quibell Bros., make quotations which secure for Kerol still further considerable advantages.

### A NEW BUILDING MATERIAL.

Apart even from the Insurance Act, the problem of building cheap sanatoria and open-air shelters is a constant one where bricks are too expensive and iron is too ugly. F. H. Heath, Limited, of 22 Bridge Street, Manchester, have produced a new type of material which is cheap, artistic, and durable. The buildings they put up are made of reinforced steel sheets and cement, the outside walls are finished with granite chippings, and the inside with patent polished plaster, while the roofs are covered with red asbestos slates interlined with inodorous felt. Messrs. Heath claim that sanatoria can be built at a cost of less than £50 per bed, and all secretaries considering the problem of extension in the matter of shelter, sanatoria, or isolation blocks could not do better than write for their specification and designs. The London address is 175 Piccadilly.

### OUR BUREAU OF INFORMATION.

Everyone who uses or wishes to help our Bureau of Information must be kind enough to observe the following rules:—

(1) Postal replies cannot be given. Exception will only be made in very special cases at the Editor's discretion.

(2) Queries or answers relating to different cases must be written, or preferably typed, on separate sheets of paper, and the writing must never be crossed.

(3) Questions and replies received not later than Wednesday will, as far as possible, be dealt with in our issue of the following Saturday week.

(4) Letters must have attached the name and address of the sender, with a pseudonym for publication if preferred.

(5) Applications referring to non-dependents where the need relates to business or employment must be accompanied by a postal order for 5s., when the requirement shall have publicity in these columns.

(6) All applicants for insertion in our list of Approved Homes must send a registration fee of five shillings. The omission of this leads to delay and gives avoidable trouble.

(7) *All communications* for this department *must be accompanied by the coupon* to be found at the bottom of the third page of the cover on the inside, and should be addressed to the Editor of the Bureau of Information, c/o The Hospital, 29 Southampton Street, Strand, London, W.C.

